# Addition of PM_**2.5**_ into the National Ambient Air Quality Standards of China and the Contribution to Air Pollution Control: The Case Study of Wuhan, China

**DOI:** 10.1155/2014/768405

**Published:** 2014-04-03

**Authors:** Mingqing You

**Affiliations:** Zhongnan University of Economics and Law, Hubei Water Affairs Research Center, 182 South Lake Avenue, East Lake High-Tech Development Zone, Wuhan 430074, China

## Abstract

PM_2.5_ has gradually become a major environmental problem of China with its rapid economic development, urbanization, and increasing of motor vehicles. Findings and awareness of serious PM_2.5_ pollution make the PM_2.5_ a new criterion pollutant of the Chinese National Ambient Air Quality Standard (NAAQS) revised in 2012. The 2012 NAAQS sets the PM_2.5_ concentrate limitation with the 24-hour average value and the annual mean value. Wuhan is quite typical among central and southern China in climate, economy, development level, and energy consumption. The data are cited from the official website of Wuhan Environmental Protection Bureau and cover the period from 1 January to 30 June 2013. The data definitely confirm the existence of serious PM_2.5_ pollution in Wuhan and indicate that the addition of PM_2.5_ as a criterion pollutant significantly brings down the attainment rate of air quality. The example of Wuhan reveals that local governments should take measures to reduce the emission of PM_2.5_ if it affects the attainment rate and the performance evaluation value of air quality. The main contribution of 2012 NAAQS is that it brings down the attainment rate of the air quality and forces local governmental officials to take the measures accordingly.

## 1. Introduction

PM_2.5_ refers to the particulate matter which is less than 2.5 *μ*m in aerodynamic diameter and is also called fine particulate matter or fine particles [1, 2]. PM_2.5_ can reduce visibility of the air and also cause health problems. It has been reported that increased PM_2.5_ concentration will make people more susceptible to certain diseases, including acute respiratory symptoms [3], asthma [4], myocardial infarction [5], and lung cancer [6]. On the opposite hand, the decrease of PM_2.5_ concentration has beneficial health effect. It has been reported that there is an association between the reduction in overall mortality and the decreased mean levels of PM_2.5_ [7]. So it is an important indicator of risks to health from particulate pollution and might also be a better indicator than the particulate matter less than 10 *μ*m in aerodynamic diameter (PM_10_) for anthropogenic produced particles in many areas [2].

PM_2.5_ gradually became a major environmental problem with the rapid economic development in China, urbanization, and increasing of motor vehicles. The GDP (gross domestic production) grows from 36.5 × 10^8^ Yuan RMB in 1978 to 47.2 × 10^12^ Yuan RMB in 2011; the average annual increase is about 9.9%; the percentage of population registered as city dwellers also grows from 17.9% in 1978 to 51.3% in 2011; the nationwide total possession of civil vehicles is increased from 1358400 units to 93563200 units; an average annual increase is about 20.87% [8]. However, air quality generally keeps a downward trend so far. Though it is reported that SO_2_ concentration in the ambient air has stopped deteriorating and is getting better in recent years [9, 10], there is no noticeable nationwide improvement as to the pollution of particulate matter. Compared with the other two indicators of particule matter, the total suspended particles (TSP) and PM_10_, PM_2.5_ is particularly problematic is the problem.

Because PM_2.5_ is not a pollution criterion in the 1996 National Ambient Air Quality Standard (1996 NAAQS) [11, 12], it was not mandatorily monitored and there is no official nationwide data as to its concentration and its percentage in the PM_10_ mass. Nevertheless, PM_2.5_ in China has attracted the attention of researchers for a long time. In 2003, Whittaker et al. found that the majority (99.7% in winter, 96.6% in summer, and 82.3% in dust storms) of the PM_10_ collected in the urban area of Beijing was in the respirable (PM_2.5_) size range [13]. Since then, many researchers reported PM_2.5_ findings, especially about the PM_2.5_ problem in large cities. Earlier findings about the PM_2.5_ problems of Beijing, Shanghai, and other large cities were summarized by Chan and Yao [11]. Among the latest published findings, Xu et al. reported the seasonal variations and chemical compositions of PM_2.5_ in the urban area of Fuzhou, China, with the data from April 2007 to January 2008 [14]; Kong et al. reported the spatial and temporal variation of phthalic acid esters (PAEs) in atmospheric PM_10_ and PM_2.5_ and the influence of ambient temperature in Tianjin, China, with the data of seven selected sites in 2010 [15]; Wang et al. reported the contamination characteristics and possible sources of PM_10_ and PM_2.5_ in different functional areas of Shanghai, China, covering the period of July 2009 through September 2010 [16].

The US embassy at Beijing and environmental nongovernmental organizations (NGOs) all join the effort to make the public better understand the detrimental effects of PM_2.5_ and the high concentration of PM_2.5_ in China, and the NAAQS of 1996 is outdated. People became more aware of the difference between their personal judgment of the air quality and the official grading of the ambient air quality based on air pollution index (API), and many people got to know that the difference is caused by the fact that PM_2.5_ was not a criterion pollutant in the 1996 NAAQS and was not taken into consideration in the calculation of API. In 2011, the issue of PM_2.5_ eventually led to the revision of NAAQS and the control of PM_2.5_ pollution [17].

So the Chinese government decided to revise the 1996 NAAQS and added PM2.5 into the new NAAQS. The Chinese Ministry of Environmental Protection (MEP) eventually issued the new National Ambient Air Quality Standard (2012 NAAQS) on 29 February 2012 [2], which replaced the 1996 NAAQS and its revision in 2000 and the Maximum Allowable Concentration of Pollutants in Atmosphere for Protecting Crops [18]. The 2012 NAAQS sets PM_2.5_ concentration limits for both the 24-hour average and the annual mean value. The 24-hour average concentration limited value is 35 ugm^−3^ for Category I places, including natural protection zones, scenic resorts, and other areas needing special protection and 75 ugm^−3^ for all other places (Category II places). The annual mean value of 15 ugm^−3^ is for Category I places and the value of 35 ugm^−3^ is for Category II places. Besides the addition of PM_2.5_ as a criterion pollutant, the 2012 NAAQS also makes other minor changes. The 2012 NAAQS will be implemented nationwide as of 1 January 2016, but the Chinese MEP is authorized to require certain places to implement it earlier. The Chinese MEP also released the new Technical Regulation on Ambient Air Quality Index (AQI) on 29 February 2012 [19], which will be implemented at the same time as the 2012 NAAQS. On 21 May 2012, the Chinese MEP first required 74 cities to monitor the air quality according to the 2012 NAAQS, give the ambient air quality with AQI, and publicly disclose the monitoring data before the end of 2012 [20].

The special contribution of this paper lies mainly in three aspects. First, it uses the latest available data generated by the official monitoring stations in the first six months of 2013 on the citywide PM_2.5_ concentration. There have been no reported findings of the PM_2.5_ data of the first 74 cities required by the Chinese MEP to monitor PM_2.5_ concentration citywide according to 2012 NAAQS. Previously published reports either were based on unofficial and experimental monitoring work or covered a limited area or within a limited period of time. Second, this paper gives more attention to the governmental policy. Previous publications paid more attention to the scientific findings than the policy aspect of PM_2.5_. Several publications reported findings on the causes of elevated PM_2.5_ concentration [21], on the spatial and temporal variation of the composition of PM_2.5_, including phthalic acid esters (PAEs) [15], polycyclic aromatic hydrocarbons [22, 23], heavy metals, and other chemicals [24]. Some other publications reported the adverse health effect of PM_2.5_, including the effect on mortality [25, 26] and COPD [27]. Only very few publications discussed policy issues [28–32]. In a government-dominated country like China, the government policy is the decisive factor for environmental protection or environmental pollution. Therefore, an important research work of environmental protection is to find how to transform scientific finding into governmental policies and study the governmental behavior towards scientific findings. Third, this paper presents in detail the implementation requirements of the 2012 NAAQS related to PM_2.5_. Some previous publications have introduced the 2012 NAAQS. Wang et al. mentioned the revised NAAQS but erroneously stated the date of adoption as 30 September 2011 instead of 29 February 2012 [16]. Tian et al. only discussed the SO_2_ and NO_*x*_ of the 2012 NAAQS, but did not mention PM_2.5_ [10]. Xue et al. estimated that the reduction of the total emission of SO_2_ and NO_*x*_ will reduce the PM_2.5_ concentration, but their data on PM_2.5_ concentration were an estimation based on an assumed ration between PM_2.5_ concentration and PM_10_ concentration [9]; the actual data are not collected by official monitoring work of a large scale. There is no other mentioning of the 2012 NAAQS on the Web of Knowledge or in other major databases.

This study takes a case approach because the large territory of China and the diversified situation make it quite hard to review the measures taken by all local governments. The case studied is in Wuhan city, one of the 74 cities which are required to monitor PM_2.5_ no later than 31 December 2012. It is chosen for this case study because it is typical and represents a large part of China (as discussed later in this paper) and also because it was rarely studied in the previous research about its air quality in general and its PM_2.5_ concentration in particular. While there is a relatively rich literature on the PM_2.5_ condition of Beijing, Shanghai, and Pearl River Delta areas [2, 11, 21, 33], Wuhan is studied as to its air pollution, while not mentioning its PM_2.5_ problem. Waldman et al. monitored PM_10_ and PM_2.5_ for 2 weeks at a residential site in Wuhan in 1988 [34]. Querol et al. reported the annual average concentration of SO_2_, NO_2_, and PM_10_ of an urban site (Hankou city) and an industrial site (Chang Qian district) [35]. Wei et al. reported short-term measurements of PM_10_ and PM_2.5_ at an urban site (Huang-Pi Jie) and a suburban site (Mo Shan) in Wuhan on selected days in four seasons during 1995 and 1996 and found that PM_2.5_ accounted for about 60% of the mass of PM_10_ [36]. These researches are either too short in temporal coverage (only two weeks [34]) or too few in monitoring sites [34, 36], and the date are too old [35].

## 2. Materials and Methods

### 2.1. Sample City Description

Wuhan city is at longitude 113°41′–115°05′E and latitude 29°58′–31°22′N. With a subtropical monsoon climate, Wuhan has a long summer time of about 135 days on average, a long winter time of almost equal length, and a short spring and a short autumn. The frost-free period is about 240 days on average. In 2011, the lowest monthly average temperature was 0.7°C of January while the highest monthly average temperature is 28.9°C of July [37].

Wuhan is the capital city of Hubei Province. It is the biggest city in central China in terms of population and area. It has a resident population of more than 10 million and a land area of 8494.41 km^2^ [37]. If water surface is included, its total area is 1286.6 km^2^. Around Wuhan, there are 8 smaller cities. These 8 cities and Wuhan form the Wuhan City Cluster.

The economy of Wuhan is in the upper middle among all megacities of China. The 2012 annual GDP of Wuhan was about RMB 8 × 10^11^ Yuan RMB or 1.31 × 10^11^ US dollars. The ratio between the first, second, and third industries was 3.8 : 48.3 : 47.9. By the end of 2012, Wuhan has 1105000 motor vehicles, including 894400 cars. On average, each one hundred households own 22.1 private cars [38].

Wuhan City Cluster is classified by the Chinese Central Government as a place to be further developed with greater efforts [39]. Wuhan is still actively seeking outside investment from other countries or other parties of China. Some polluting industries or companies are moving to Wuhan from more prosperous coastal areas of China where local governments enforce environmental law more stringently. This makes Wuhan different from Beijing, Shanghai, and Pearl River Delta. Wuhan currently is in a boom of housing and road projects. In the year 2012, buildings under construction were of 68629700 m^2^ in terms of the floor space [38]. Besides, several roads were also under construction.

Wuhan cannot meet its electricity consumption completely only with hydroelectricity. In 2011, 55.72% of the electricity demand was met with the electricity generated by local thermal power plants. The four most important energy sources for enterprises are coal, crude oil, coke, and electricity. After converting into standard coal equivalent (SCE), coal, crude oil, coke, and electricity account for 23.38%, 16.38%, 15.21%, and 6.14%, respectively, of the total energy consumption by enterprises in 2011 [37]. For domestic life and third industries, the most important energy source is electricity. Gas is used for cooking. Virtually no coal is used for domestic life or third industries. Like many cities in the central and southern China, Wuhan does not have a citywide centralized heating system.

Wuhan is within the acid rain control area demarcated by the former State Administration of Environmental Protection in 1998 (SEPA, 1998). There was a general decrease of SO_2_ concentration in the ambient air over the past years. In 2012, the total discharge of SO_2_ was 105800 tons and the annual average of SO_2_ in the ambient air is 0.030 mg m^−3^ [40]. Besides SO_2_, Wuhan also suffers NO_*x*_ pollution.

Wuhan city is quite typical among central and southern China in climate, economy, development level, and energy consumption. So the case study of Wuhan city not only can reveal the situation of Wuhan but also can reflect a large area in central and southern China, which makes Wuhan city a suitable sample.

### 2.2. Sources of Data on PM_**2.5**_ Concentration

**Figure 1:**
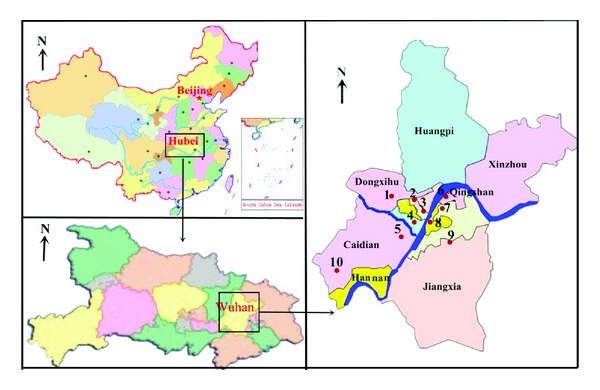
Map of Wuhan with districts and national-level ambient air monitoring stations.

The data of PM_2.5_ and other pollutants of Wuhan are cited from the official website of Wuhan Environmental Protection Bureau (Wuhan EPB) (http://www.whepb.gov.cn) and cover the period from 1 January to 30 June 2013. Wuhan EPB maintains 10 national-level monitoring stations for air pollutants (see Figure 1 and Table 1). These monitoring stations use the gravimetric method to determine the concentrations of PM_10_ and PM_2.5_ [41]. Among these 10 monitoring sites, Chenhu Qihao is within a wetland protection zone, 40 km away from the third ring road. It functions as the only control sample reflecting the quality of background ambient air and its data is not used to calculate the city-wide average concentration. The monitoring results of the other 9 monitoring stations are averaged into the city-wide air quality data. Wuhan EPB's website reports on a daily basis the data of these 10 monitoring stations as well as the citywide data. The real-time data, with a time resolution of one hour, of these 10 monitoring stations and the citywide average are also available on the Chinese MEP's official website (http://www.mep.gov.cn).

### 2.3. Coverage of Policies Reviewed

This paper introduces the governmental measures taken by the government at the national, provincial, and city levels during the period from the adoption of the 2012 NAAQS to 30 June 2013. Since the Communist Party of China (CPC) is the leading political party in China and has the dominant influence on the Chinese government, this paper also reviews relevant policies of the CPC.

## 3. Results and Discussion

### 3.1. The Citywide Average Concentration of PM_**2.5**_

The Technical Regulation on Ambient Air Quality Index (on trial) issued by the Chinese MEP provides for 8 brackets of the 24-hour average PM_2.5_ concentration and sets index values for each bracket. It also classifies the index values into six grades, from Grade I (excellent) to Grade VI (extremely polluted). Grades I and II are attainment grades while all others nonattainment. [19] The attainment status, brackets, and grades provided in this technical regulation are useful terms to describe the PM_2.5_ concentration.

In only 7 days, the 24-hour average PM_2.5_ concentration was at or below 35 ug m^−3^ (Grade I). In 40 days, the 24-hour average concentration was more than 35 but not more than 75 ug m^−3^ (Grade II). As 75 ug m^−3^ is the attainment limited value for the 24-hour average concentration of PM_2.5_, there were altogether 47 attainment days. All other days were nonattainment days, with various degrees of seriousness. The number of days of each grade clearly demonstrates the seriousness of PM_2.5_ pollution in Wuhan during the first 6 months of 2013 (see Table 2). The official monitoring results definitely confirm the previous studies that Wuhan had serious PM_2.5_ pollution.

### 3.2. PM_**2.5**_ as the Most Important Primary Pollutant

According to the Technical Regulation on Ambient Air Quality Index (on trial), except when the air quality as a whole is Grade I, the criterion pollutant with the highest individual air quality index shall be listed as the primary pollutant on the air quality report. Among the 181 days from 1 January to 30 June of 2013, only in 10 days (5 Feb., 19 Feb., 8 May, 16 May, 26 May, 7 June, 8 June, 16 June, 25 June, and 28 June), or 5.52%, all criteria pollutants met Grade I and the air quality as a whole was Grade I. In all other 171 days, or 94.48%, at least one pollutant exceeded the limits of Grade I. PM_2.5_ was the primary pollutant for most of these 171 days. This indicates that PM_2.5_ is the most problematic pollutant in Wuhan (see Table 3). On 8 March 2013, both PM_2.5_ and PM_10_ are listed as the primary pollutants as they had the same index value.

### 3.3. Contribution of PM_**2.5**_ to the Nonattainment of the Air Quality

Under the 2012 NAAQS, PM_2.5_ is only one criterion pollutant. Besides it, there are 5 other criteria pollutants: SO_2_, NO_2_, CO, O_3_, and PM_10_. The daily AQI is based on the individual air quality index (IAQI) of 7 monitored indicators: the 24-hour average concentration of SO_2_, NO_2_, CO, PM_10_, PM_2.5_, the highest one-hour average concentration of O_3_, and the highest 8-hour average concentration of O_3_. If the overall AQI is higher than 100 on a particular day, that day is a* nonattainment* day. If the IAQI is higher than 100 for a particular pollutant, that pollutant is the* nonattainment* pollutant.

The percentage of attainment days, or days with an AQI at or below 100, in the first 6 months of 2013 was quite low. The month with the lowest attainment rate (3.2%) was January while the months with the highest attainment rate (66.67%) were April and June.

**Figure 2:**
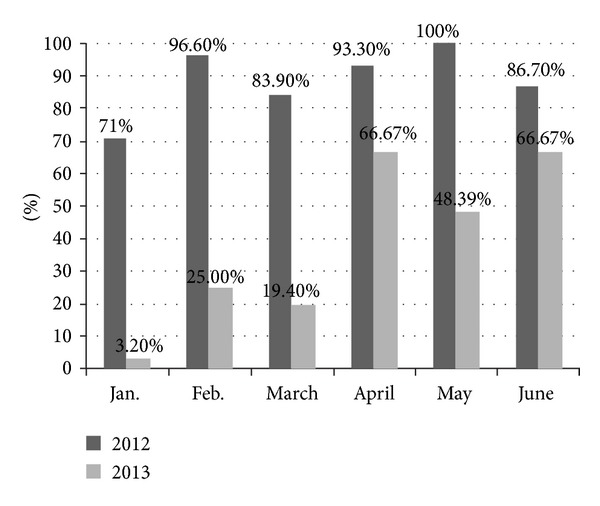
Comparison of the attainment rates of the citywide air quality during the first 6 months of 2012 and 2013.

This is a sharp contrast with the high attainment rates in the corresponding period of 2012 when the 1996 NAAQS was applicable.  Figure 2 is the comparison of the attainment rates of the first 6 months of 2013 with the corresponding months of 2012. The information of the monthly attainment rates of 2012 is taken from the corresponding monthly environmental reports issued by the Wuhan EPB on its official website.

PM_2.5_ is the leading factor in reducing attainment rate. In order to determine the contribution of PM_2.5_ to the high nonattainment rate, this paper compares the number of days when PM_2.5_ was the nonattainment pollutant, that is, the days when the 24-hour average concentration of PM_2.5_ exceeded the limit of 75 ug m^−3^ or the IAQI of PM_2.5_ exceeded 100, with the number of overall nonattainment days, that is, the days when the overall AQI exceeded 100. The contribution rate is the number of PM_2.5_ nonattainment days divided by the number of overall nonattainment days. In the first 4 months, the contribution rate of PM_2.5_ to the nonattainment was 100%. Every day when the overall air quality failed to attain the prescribed standard, the 24-hour average concentration of PM_2.5_ also exceeded the prescribed limit. The contribution rate of PM_2.5_ is decreased in May. In 9 out of the 16 overall nonattainment days of May 2013, the 24-hour concentration of PM_2.5_ also exceeded the prescribed limit. For the remaining 7 days of overall nonattainment, the nonattainment pollutant was O_3_. The situation of June was more complex. There were 11 days in June 2013 when the IAQI of PM_2.5_ exceeded 100. But on 3 days (6 June, 10 June, and 23 June 2013), the IAQI of other pollutants were low and brought down the overall AQI into the range of attainment. However, because of the high IAQI of O_3_, the overall AQI exceeded 100 on 13 June and 20 June 2013 though the IAQI of PM_2.5_ was less than 100. Overall, PM_2.5_ made a high contribution to the high nonattainment rate (Table 4), excluding the 3 days (6 June, 10 June, and 23 June 2013) when the overall AQI was blow 100.

### 3.4. Politics behind the Addition of PM_**2.5**_ as a Criterion Pollutant

China has a centralized unitary political system. There is no division of legislative power between the national government and the provincial or other local governments. Theoretically, all local governments, including the local governments of autonomous regions, prefectures, or counties where people of minority nationalities account for a significant percentage, should follow the legislations and requirements of the national government. This gives people the impression that whatever the national government decides, either in the form of laws by the legislative branch or in the form of governmental plans and administrative orders by the executive branch, the local governments will follow. But the actual situation is far more complex.

Like the government of all other countries, the Chinese government is also suffering from agency problems. Governmental officials are only agents of the government. Without incentives, governmental officials tend to do less to minimize their personal risks and maximize their personal benefits. As to governments highly accountable to voters, the public opinion of the voters is the key factor incentivizing governmental officials. In China, the opinion of the voters is a much less direct and powerful incentive to local governmental officials than the opinion of the political power at higher levels, because the political power at the higher level decides the promotion of governmental leaders at lower levels. The higher government maintains a performance evaluation system to evaluate leaders of its subordinate lower governments, and this evaluation is an important factor for the promotion of governmental officials. In such a political system, the attainment of performance indicators is in fact more important than the achievements to be evaluated with performance indicators.

In the past years since the late 1970s, the economic development was the central element of the performance evaluation of lower governments and the GDP was the key performance indicator for the evaluation of economic development. The leaders of many local governments utilized all available political and economic resources to drive up local GDP. This led to a serious distortion of the local public policy. Environmental interests in many places were sacrificed for the short-term economic development. This not only hurt the environment and the public health but also hurt the long-term economic development [42–44].

As the national government gave more and more attention to environmental protection in recent years, attainment of environmental protection tasks gradually becomes more and more important. The Environmental Protection Law of China provides in Article 16 that the local governments at various levels shall be responsible for the environment quality of areas under their jurisdiction and take measures to improve the environment quality. Just like GDP to the economy, attainment rate is the indicator of the performance in environmental protection. To a large extent, the responsibility of the local governments for environment quality is transformed into the attainment of environmental protection indicators set by the higher government.

The NAAQS directly affects the attainment rate of local governments in their task of air pollution control. This political situation also makes its hard to upgrade NAAQS. The Chinese MEP has realized the elevation of the PM_2.5_ concentration in the ambient air with the development of economy in the past years and considered adding PM_2.5_ as a pollution criterion. However, local governments were afraid that upgrading the NAAQS, especially adding PM_2.5_ as a criterion pollutant, would make it harder to achieve attainment goals and harder to keep the speed of economic development, which is also an important criterion in the performance evaluation of local governmental leaders. The MEP had no sufficient political resources to add PM_2.5_ as a new criterion pollutant and upgrade the NAAQS; the final decision has to be made by the State Council, the cabinet of the Chinese national government.

### 3.5. Policy Measures to Enhance Attainment by the Chinese National Government

The control of air pollution is put on an ever high position in the political agenda of the Communist Party of China (CPC). In the Eighteenth CPC National Congress held in 2012, ecological civilization was put alongside with economic, political, cultural, and social development to form a five-in-one overall development plan, for the purpose of leading to increased production, prosperity, and a good ecosystem. In this conference, it was expressly requested to “take a holistic approach to intensifying prevention and control of water, air and soil pollution, putting prevention first and placing emphasis on serious environmental problems that pose health hazards to the people” [45].

The China MEP proposed 5 key tasks in the near future in the notice issued on 29 February 2012 to implement the 2012 NAAQS: (1) research and attainment plan: environmental protection agencies should establish an inventory of air pollution sources and carry out relevant scientific research to provide better technological support for the control of air pollution and important nonattainment cities should make attainment plan and submit it to higher governmental authority for approval; (2) enhancement of the environmental requirements on market accession: the development of pollution industries and the export of their products should be strictly controlled; (3) joint prevention and control of air pollution in important regions: local governments of Beijing-Tianjin-Hebei region, Yangtze River Delta, and Pearl River Delta should take joint measures to prevent and control air pollution; (4) control on air pollution from motor vehicles: the government should make use of economic incentive as well as command-and-control measures to improve the standard of fuels and motor vehicles, including the phasing out of substandard in-use motor vehicles; (5) monitoring and precautionary reports of air pollution episodes: environmental protection agencies at or above prefecture level should make real-time and daily reports of air quality according to the requirements on AQI, make contingency plans for air pollution episodes, and respond to foreseen episodes by giving warnings to the public and taking measures to reduce the discharge of air pollutants, including reduction of production and air pollutant discharge of key sources of pollution, suspension of civil engineering projects, and restriction on motor vehicles [46]. Among these 5 key tasks, joint prevention and control of air pollution in important regions is a newly adopted innovative regulatory measure. It requires the horizontal cooperation among local governments. It affects the horizontal relationship among local governments as well as the vertical relationship between local governments and their common superior government. As PM_2.5_ not only causes local pollution but also can be carried to places far away and cause pollution, this measure may be vital for the control of PM_2.5_ pollution. How this measure actually functions is yet to be further studied with more empirical data. It suffices here to say that this measure makes local governments keep an eye on their neighbors.

The MEP, the National Development and Reform Commission (NDRC), and the Ministry of Finance (MOF) issued the Plan for Prevention and Control of Atmospheric Pollution in Key Regions During the Period of the Twelfth Five-year Plan on 6 October 2012 [47]. This plan sets air pollution goals for 13 key regions, including Wuhan City Group. Annual mean of PM_2.5_ concentration is one of the goals. Other goals include the annual mean of SO_2_, NO_2_, and PM_10_, the emission of industrial dusts, and the VOCs emission from in-use sources of key industries. As to Wuhan City Group, this plan requires a 5% reduction of the annual mean of PM_2.5_ concentration by the end of 2015.

As a further measure to control air pollution of the key regions provided in the Plan for Prevention and Control of Atmospheric Pollution in Key Regions During the Period of the Twelfth Five-year Plan, the MEP decided to implement the special emission limitation for air pollutants on 27 February 2013 [48]. The special emission limitation is a new regulatory tool. It is a more stringent pollution emission standard applicable to specified industries of specified regions. To make it work, the MEP first provides the special limitation in relevant emission standards, and then the MEP may decide when and where to implement such special emission limitations. This regulatory tool was first used for the control of water pollution. The Emission Standard of Pollutants for Sulfuric Acid Industry is the first emission standard providing for a special emission limitation for air pollutants [49]. During the twelfth five-year plan period (2010–2015), the special emission limitation for air pollutants will be applicable to thermal power plants, steel and iron industry, petrochemical industry, cement, nonferrous metal industry, and chemical industry in the key regions.

The Chinese MEP also issued technical policies to control air pollution from certain industries, including cement industry, steel and iron industry, sulfuric acid industry, and VOCs. These technical policies provide for the requirements on technical innovation, economic incentives, and other issues. They do not only address PM_2.5_ pollution but also have much positive effect on the reduction of PM_2.5_ concentration.

The measures of the MEP were endorsed by the State Council. In a State Council meeting held by Premier Li Keqiang, these measures were summarized into 10 points. The above measures taken by the national government indicate the strong political will of the national government to control air pollution, particularly the PM_2.5_ pollution. The national government noticeably changed its attitude towards the control of air pollution, particularly PM_2.5_ pollution, since the adoption of the 2012 NAAQS. This can be explained that the national government of China is ultimately responsible for the legitimacy of the government and accountable to the people. As the concentration limits are already provided in the 2012 NAAQS, the national government needs to take effective measures to honor the newly adopted NAAQS by increasing the attainment rate. To the national government, attainment is not only for environmental protection but also more importantly, for the governmental credibility. This puts the attainment of the 2012 NAAQS at a high position in the political agenda of the national government. Accordingly, the national government took measures to reduce PM_2.5_ concentration and request local governments to act.

### 3.6. Reaction of the Local Government of Hubei Province

The Hubei Provincial Commission of the CPC followed the CPC Central Commission and gave similar support for the control of air pollution. In the 10th representative conference of the Hubei Provincial Commission of the CPC held on 9 June 2012, safe drinking water, air pollution, and soil pollution were listed as main tasks of environmental protection work [50].

The Outline of the Hubei Provincial Environmental Protection Plan during the Period of the Twelfth Five-year Plan expressly requires the reduction of PM_2.5_ concentration. For this purpose, it encourages the development of clean energy, promotes further reduction of industrial dust, and emphasizes the control of fugitive discharge of air pollutants. This plan also requires the government to conduct pioneer research projects on the current conditions of PM_2.5_ pollution and causes of hazy days of Wuhan City Cluster, to investigate sources of air pollution of the whole province, and to take appropriate measures for point sources and fugitive sources of air pollutants [51].

The government of Hubei Province also promoted the cooperation between the environmental protection authorities and meteorological authorities. The Environmental Protection Bureau of Hubei Province (Hubei EPB) signed a long-term cooperation agreement with the Meteorology Bureau of Hubei Province in June 2013 on monitoring, prewarning, and forecasting of air quality. Under this agreement, the two authorities will establish a communication and coordination mechanism, share information, jointly establish monitoring facilities, establish integrated information disclosure platform, jointly investigate and evaluate serious environmental pollution incidents, make joint contingency plans for serious haze episodes, consult with each other before issuing prewarning to the public, and cooperate on other related issues. This interdepartmental cooperation will enhance the ability to predict the high concentration of PM_2.5_ and implement contingency plans.

In addition, Hubei Province also took more stringent measure to control the emission of SO_2_, NO_*x*_, and VOCs. These measures are mainly for the attainment of reduction goals on SO_2_ and NO_*x*_ set by the national government but will make positive contribution to the attainment of PM_2.5_ concentration.

### 3.7. Reaction of the Local Government of Wuhan

The Wuhan government enlarged the prohibitive and restrictive areas of high-polluting fuels in March 2012. Facilities using high-polluting fuels within the prescribed area will gradually be phased out. After 1 January 2015, it will be prohibited to sell or use high-polluting fuels in the prohibitive area [52].

The Environmental Protection Bureau of Wuhan (Wuhan EPB) took several measures to reduce PM_2.5_ concentration. It made a plan on 26 March 2013 to upgrade and/or phase out boilers powered with coal in the area between the second and third ring roads. It is implementing this plan currently. About 360 heavy duty boilers are expected to be either upgraded or phased out [53].

The local contingency plan also made due consideration of serious air pollution episodes. The government of Wuhan city issued on 19 March 2013 the General Contingency Plan for Incidents of Wuhan City [54]. This general contingency plan takes thick fog and haze as one form of incidents and impose on Wuhan EPB the principal duty to take contingency measures. Accordingly, Wuhan EPB drafted its departmental contingency plan for serious air pollution episodes, which was approved by the government of Wuhan city [55]. This contingency plan provides for restrictive measures on motor vehicles, industrial production, and civil engineering in days of high AQI values. As PM_2.5_ is the most problematic air pollutant of Wuhan, these contingency measures are most likely to be taken when the PM_2.5_ concentration is high.

Prohibition of firecracker is the most recent measures taken by Wuhan to reduce PM_2.5_ concentration. The People's Congress of Wuhan revised a local regulation in June 2006 to allow the sale and use of firecrackers in urban areas during a limited number of days before and after the Chinese Lunar New Year. Firecrackers cause noises as well as serious air pollution, especially PM_2.5_ pollution. The Wuhan government recently submitted a draft to the local people's congress to prohibit the sale and use of firecrackers in all central districts and urbanized areas of other districts. This draft will soon be adopted by the local people's congress.

The abovementioned measures taken by Hubei Province and Wuhan city indicate that the attainment rate is a strong incentive for local governments. Once PM_2.5_ is added into the NAAQS and affects the attainment rate, local governments will act.

## 4. Conclusion

The latest available official monitoring data on the concentration of PM_2.5_ confirms that there is serious PM_2.5_ pollution in Wuhan. As many mega-cities have a similar situation as Wuhan, it is very likely that there is serious PM_2.5_ pollution nationwide. Before PM_2.5_ was added as a pollution criterion in the NAAQS, the air quality attainment rate was high at Wuhan in 2012 under the 1996 NAAQS. As PM_2.5_ is the most problematic air pollutant to Wuhan, the addition of PM_2.5_ as a criterion pollutant in the 2012 NAAQS greatly brought down the air quality attainment rate of Wuhan when the 2012 NAAQS became applicable. Both the MEP and local governments knew this would happen. Because of the strong political opposition from local governments, the MEP did not have sufficient political resources to upgrade the NAAQS. Eventually the concern about governmental credibility overrid the concern about economic development and attainment rate, and PM_2.5_ was added as a criterion pollutant to the 2012 NAAQS more for governmental credibility than for the public health. As the national government is ultimately responsible for governmental legitimacy and credibility, it took measures to honor the 2012 NAAQS and reduce PM_2.5_ concentration. In a political system where the local governmental officials are more accountable to the higher political power, local governmental officials care more about the attainment rate, which is part of the performance evaluation, than the environmental protection itself. The performance evaluation is an important incentive for local governmental officials to take measures to reduce PM_2.5_ concentration. As the example of Wuhan reveals, local governments would take measures to reduce PM_2.5_ concentration if it affects the attainment rate and the performance evaluation of governmental officials. The main contribution of 2012 NAAQS to air pollution control is that it brings down the attainment rate and forces local governmental officials to act. This conclusion may also be applicable to other environmental quality standards of China, and further research is also necessary.

## Figures and Tables

**Table 1:** National-level ambient air monitoring stations of Wuhan.

Number	Name	Description
1	Wujiashan	Wujiashan Middle School, Dongxihu District
2	Hankou Huaqiao	Huaqiao Primary School (Huaqiao Second Village Division), Jiangan District
3	Hankou Riverside	Riverside Municipal Square, Jiangan District
4	Hanyang Yuehu	Yuehu Lake Garden, Qintai Road, Hanyang District
5	Zhuankou Xinqu	Public Health Service Center, Wuhan Economic and Technical Development Zone
6	Qingshan Ganghua	1250 Heping Avenue, Qingshan District (China Metallurgical Geology Bureau, Zhongnan Sub-Bureau)
7	East Lake Liyuan	Liyuan, East Lake Ecological Tourism Resort
8	Wuchang Ziyang	198 Shouyi Road, Wuchang District (Culture and Sports Bureau of Wuchang District)
9	East Lake High-Tech	11 Huashiyuan North Road, East Lake High-Tech Development Zone (Hongyu Environmental Protection Technological Garden)
10	Chenhu Qihao	Qihao Village, Xiaosi Township, Caidian District

**Table 2:** City-wide 24-hour average PM_2.5_ concentrations from 1 January to 30 June 2013.

Attainment status	Grades	Brackets of 24-hour average PM_2.5_ concentration	Number of days	Percentage	Attainment and nonattainment rate
Attainment	I	[0, 35)	7	3.87%	25.97%
	II	[35, 75)	40	22.10%	

Nonattainment	III	[75, 115)	53	29.28%	74.03%
	IV	[115, 150)	26	14.36%	
	V	[150, 250)	36	19.89%	
	VI	[250, 350)	16	8.84%	
		[350, 500)	3	1.66%	
		[500, +*∞*)	0	0.00%	

Total	—	—	181	100%	100%

**Table 3:** Breakdown of the number of days in terms of primary pollutants.

Primary pollutant	Number of days	Percentage
PM_2.5_	125	73.10%
PM_10_	16	9.36%
O_3_	27	15.79%
NO_2_	6	3.51%
Total	**171** ^ a^	**100%** ^ a^

^a^On 8 March 2013, both PM_2.5_ and PM_10_ are listed as the primary pollutants as they had the same index value.

**Table 4:** Contribution of PM_2.5_ to the nonattainment rates of the first 6 months of 2013.

Months	Overall nonattainment days	PM_2.5_ nonattainment days	Contribution rate
January	30	30	100%
February	21	21	100%
March	25	25	100%
April	10	10	100%
May	16	9	56.25%
June	10	8^a^	80%
Total	**102**	**103**	**91.96%**

^a^On 8 March 2013, both PM_2.5_ and PM_10_ are listed as the primary pollutants as they had the same index value.
